# Explaining the variation in the management of lifestyle risk factors in primary health care: A multilevel cross sectional study

**DOI:** 10.1186/1471-2458-9-165

**Published:** 2009-05-29

**Authors:** Rachel A Laws, Upali W Jayasinghe, Mark F Harris, Anna M Williams, Gawaine Powell Davies, Lynn A Kemp, The Community Health SNAP Project Team

**Affiliations:** 1 Centre for Primary Health Care & Equity, School of Public Health and Community Medicine, University of New South Wales, Sydney NSW 2052, Australia

## Abstract

**Background:**

Despite evidence for the effectiveness of interventions to modify lifestyle behaviours in the primary health care (PHC) setting, assessment and intervention for these behaviours remains low in routine practice. Little is known about the relative importance of various determinants of practice.

This study aimed to examine the relative importance of provider characteristics and attitudes, patient characteristics and consultation factors in determining the rate of assessment and intervention for lifestyle risk factors in PHC.

**Methods:**

A prospective audit of assessment and intervention for lifestyle risk factors was undertaken by PHC nurses and allied health providers (n = 57) for all patients seen (n = 732) over a two week period. Providers completed a survey to assess key attitudes related to addressing lifestyle issues. Multi-level logistic regression analysis of patient audit records was undertaken. Associations between variables from both data sources were examined, together with the variance explained by patient and consultation (level 1) and provider (level 2) factors.

**Results:**

There was significant variance between providers in the assessment and intervention for lifestyle risk factors. The consultation type and reason for the visit were the most important in explaining the variation in assessment practices, however these factors along with patient and provider variables accounted for less than 20% of the variance. In contrast, multi-level models showed that provider factors were most important in explaining the variance in intervention practices, in particular, the location of the team in which providers worked (urban or rural) and provider perceptions of their effectiveness and accessibility of support services. After controlling for provider variables, patients' socio-economic status, the reason for the visit and providers' perceptions of the 'appropriateness' of addressing risk factors in the consultation were all significantly associated with providing optimal intervention. Together, measured patient consultation and provider variables accounted for most (80%) of the variation in intervention practices between providers.

**Conclusion:**

The findings highlight the importance of provider factors such as beliefs and attitudes, team location and work context in understanding variations in the provision of lifestyle intervention in PHC. Further studies of this type are required to identify variables that improve the proportion of variance explained in assessment practices.

## Background

Behavioural risk factors such as smoking, poor nutrition, at-risk alcohol consumption and physical inactivity are the main preventable risk factors for chronic conditions which account for more than 60% of the overall global burden of disease now, and an expected 80% by the year 2020 [1]. Primary health care (PHC) has been identified as a suitable setting for interventions to reduce behavioural risk factors due to contact with the general population and continuity of care which provide opportunities for risk factor assessment, brief intervention and referral to support services or programs [2]. There is growing evidence that brief interventions for behavioural risk factors delivered in PHC can be effective, particularly for smoking cessation and problem drinking [3-5]. Despite this, levels of intervention in routine practice remain low [6,7], highlighting the need for a better understanding of the range of factors influencing the management of behavioural risk factors in PHC practice [8].

A number of studies have explored factors influencing the management of lifestyle issues in PHC, mainly through the cross sectional analysis of factors associated with self reported practice in provider surveys. These studies have reported associations between a range of provider and organisational factors and the management of behavioural risk factors including provider characteristics (age, gender, provider type) [9-11], beliefs and attitudes (in particular confidence to intervene and perceived effectiveness) [12-16], work context (eg size or location of practice), and system barriers such as lack of time and financial incentives [10,14,17]. Other studies have examined correlates of risk factor management practices as reported by patients or noted in direct observation of consultations. These studies have reported differences in recall or observation of advice provision for lifestyle risk factors according to the patients' gender [18-23], age [18-21,24], socio-economic status [18,20,22,24,25], number of existing conditions and risk factors [19,20,26] and primary care attendance rates [20].

From the available published evidence it is difficult to ascertain the relative importance or impact the various factors have on the uptake of behavioural risk factor management by PHC providers. A few studies have examined patient and practitioner characteristics associated with providing alcohol intervention through audits of individual patient medical records [27-30]. These studies however did not examine the impact of visit or consultation factors or provider beliefs and attitudes about risk factor interventions. Such studies have also rarely used multi-level analysis to account for clustering of outcomes by provider and to determine the proportion of variability attributable to each level of analysis (patient and provider)[31]. Only one of the studies that employed multi-level analysis reported the proportion of variability attributable to each level of analysis [32]. This study reported that approximately 20% of the variance in cardiovascular prevention activities between general practitioners (GP) was attributable to patient level factors and 38% to GP level factors such as workload and practice location [32]. Thus the relative importance of provider, patient and contextual factors in influencing the management of behavioural risk factors remains largely under-explored. This limits general understanding of how to intervene to reduce the evidence practice gap in the management of behavioural risk factors.

To address this gap in knowledge, this study aimed to examine the relative importance of provider, patient and consultation factors in influencing the management of lifestyle risk by PHC providers using multi-level analysis.

## Methods

This paper presents findings from a cross sectional analysis of baseline behavioural risk factor management practices for three community health teams participating in a feasibility study to develop and test approaches to integrate lifestyle risk factor management into routine practice. The study focused on the four risk factors of smoking, nutrition, alcohol and physical activity (SNAP) as these are the main behavioural risk factors for chronic disease and have common approaches for assessment and management [2].

### Description of Participating Teams/Services

The project involved three community health teams from two Area Health Services (AHS) in the state of New South Wales (NSW), Australia. In NSW, AHS are responsible for providing all hospital and community based health care apart from general practice and PHC services for specific population groups such as Aboriginal Medical Services which are funded by the Commonwealth Government. Community health services are the second largest provider of publicly funded PHC services to the general population after general practitioners (GPs) [33].

All eight AHS in NSW were invited to express interest in participating in the study and to nominate suitable teams. A total of three Community health teams were selected from two of three AHS who expressed interest. Selection was based on the capacity of the team to be involved and the relevance of risk factor management to the type of service provided and health care context. Teams were also selected to maximise the variability in team characteristics including provider type, team location (co-located or not), geographical locality, management structures and health system context.

Team one (n = 35) was a generalist community nurse team with both enrolled and registered generalist community nurses, located in a metropolitan area. Team two (n = 16) was a co-located multi-disciplinary community health team from a rural area. This team consisted of generalist community nurses, child and family nurses and allied health staff. Team three (n = 10) consisted of PHC nurses, Aboriginal health workers, and allied health practitioners providing PHC services to rural and remote communities that generally did not have access to other health services such as a GP (see appendix 1 for a description of the role of the various providers involved in the project).

### Prospective Audit of Risk Factor Management Practices

As part of a baseline assessment of risk factor management practices, all providers were asked to complete a paper based audit of risk factor management activities undertaken for each patient seen during a two week audit period. For each patient seen during the audit period, providers recorded whether they had assessed for and provided intervention in the form of verbal or written advice or a referral for the four lifestyle risk factors (smoking, nutrition, alcohol and physical activity). Providers could also indicate if they had provided an intervention to the patient in a previous consultation (in the case of review visits). Assessment was defined as having asked the patient sufficient information to determine whether the risk factor was present or not. Providers recorded patient characteristics including age, gender and postcode of residence, as well as consultation variables such as the reason for the visit (open response) and type of visit (first or follow up). Providers indicated on the audit record whether they planned to discuss risk factors prior to the consultation (yes/no) and whether it was appropriate to address risk factors with the patient (and if not appropriate the reason why, provided as an open response). The provider was asked to complete the audit record as soon as possible after each consultation.

### Outcome (Dependent) Variables

The two outcomes of interest were 1) whether the patient had been assessed for lifestyle risk factors and 2) whether patients identified to have a SNAP risk factor had received intervention (in the form of verbal advice, written advice and/or referral) during the current visit or in a previous consultation. Due to the large number of possible dependent variables (8 in total: assessment and intervention for each risk factor) preliminary analysis was undertaken to determine whether it would be possible to create one outcome variable for assessment and one outcome variable reflecting intervention practices. In terms of assessment variables, a chi square analysis was undertaken to look at the relationship between assessment for smoking and alcohol and between assessment for nutrition and physical activity (see additional file 1). This analysis suggested that these variables were significantly related and thus could be summed to create an aggregate assessment score across all risk factors. The distribution of the resulting variable was highly skewed and for the purpose of multi-level logistic regression analysis was recoded into a dichotomous variable with 0 = assessment for three or less risk factors (sub-optimal practice, n = 336, 45.9%) or 1 = assessment for all four risk factors (optimal practice, n = 396, 54.1%).

Intervention practices were firstly examined by risk factor to determine the number and proportion of patients with each risk factor who were recorded to have received an intervention. For the purposes of analysis intervention practices were dichotomised with 0 = providing intervention for none or only some of the lifestyle risk factors (sub-optimal practice, n = 42, 13.7%) or 1 = providing intervention for all lifestyle risk factors identified (optimal practice, n = 265, 86.3%). The rationale for not examining the risk factors individually was two fold. Firstly there were not sufficient numbers of cases per risk factor to allow multi-level analysis, particularly given the few 'at-risk' individuals who did not receive an intervention and the large number of independent variables. Secondly, in practice individuals often present with more than one risk factor. The definition of optimal and sub-optimal assessment and intervention practices used is in line with best practice guidelines that suggest that providers should attempt to assess and provide brief intervention for all lifestyle risk factors [2].

### Independent (Explanatory) Variables

The first set of independent variables were patient characteristics including age (collapsed into three categories: 18–44, 45–64 and 65+ years), gender and the 2006 index of relative socio-economic advantage/disadvantage for the area in which the patient lived [34]. The index ranks geographical areas where a high proportion of people are relatively more, or less, disadvantaged taking into account income, education, occupation, wealth and living conditions. A lower score indicates that an area is relatively disadvantaged compared to an area with a higher score [34] The index was linked to the patients' postcode of residence using quintiles. A quintile number of one represented the lowest 20% of areas, up to the highest 20% of areas which were given a quintile number of five. For the purposes of analysis three categories were created: 1) most disadvantaged patients (quintiles one and two), 2) intermediate disadvantaged patients (quintile three) and 3) least disadvantaged patients (quintiles four and five).

Consultation characteristics were also included as independent variables. The first of these variables was the main reason for the visit or consultation. This was provided as an open response on the audit record and was subsequently coded into one of eight main categories (PHC clinic, wound management, procedures (such as medication administration or catheterisation), chronic disease care, palliative care, allied health, child and family and support) for analysis. When examining intervention practices these categories were further collapsed into three groups 1) wound management, procedures and PHC clinics (PHC focus), 2) palliative care and 3) other reasons (specialist focus including child health and allied health consultations). The collapsing of similar categories together was required because of the smaller number of cases included in the intervention models that only focused on 'at risk' individuals.

Providers also recorded the type of consultation (first or review consultation) and whether they thought it was appropriate to address lifestyle risk factors in the consultation (yes/no) and if no the reasons why (open response). Open responses were subsequently coded into four main categories including not appropriate due to: 1) extrinsic patient or service factors (such as palliative care, physically unable to respond, frail aged, incapacitated, mental health problems, intellectual disability, non English speaking, lack of time, discrete/casual service, ongoing care), 2) perception of low patient acceptability (inappropriate time, patient not interested, religious beliefs), 3) no risk factor present and 4) risk factor previously addressed. The providers who recorded that it was not appropriate to address risk factors as either no risk factor was present or risk factors were previously addressed had assessed the risk factor in the current or previous consultation. As these responses are highly correlated with the outcome 'assessment' this variable was excluded from the assessment model. For the intervention model the two 'not appropriate' categories were collapsed resulting in a dichotomous variable: 1) appropriate to address or 2) not appropriate to address (due to extrinsic patient/service factors or perception of low patient acceptability).

A number of provider factors were also included as independent variables. Most of these variables were collected as part of a baseline survey and linked to the audit data through the use of a single identification code for each provider. Details of the survey design and administration have been described elsewhere [12]. Variables included whether the provider had planned to discuss risk factors prior to the consultation (yes/no), the team in which the provider worked (team 1, 2 or 3) and provider type (registered nurse, enrolled nurse or allied health practitioner). Due to the smaller number of cases in the intervention model the team variable was collapsed into two categories: 1) urban (team one) and 2) rural (teams two and three) and provider type into two categories: 1) nurses (registered and enrolled) and 2) allied health practitioner. Provider gender was not included as an independent variable as all providers were female, with the exception of four males. Other provider characteristics such as age and years of experience were not included in the multi level analysis as these factors were not associated with assessment or intervention in a cross sectional analysis of the survey data within the feasibility study [12].

Provider attitude measures collected as part of the baseline survey were included in the multi-level analysis and treated as continuous independent variables. These included provider ratings of perceived work priority for risk factor assessment and management, perceived acceptability of raising risk factor issues with patients and perceived level of effectiveness and the accessibility of support services. All attitude items were measured for each risk factor (smoking, nutrition, alcohol and physical activity) on a 5 point Likert scale. Each variable consisted of a mean of four items (one item for each SNAP risk factor) to create a single score for each attitude variable by provider. For example work priority rating consisted of the mean score for work priority ratings given for smoking, nutrition, alcohol and physical activity. In the case of missing data, the mean was calculated when two or more scores were present, otherwise the variable was considered missing for that case. The calculation of aggregate attitude variables across risk factors was supported by principle component analysis which suggested that items for each risk factor loaded onto one factor (results not presented).

### Statistical Analysis

The patient audit data was initially subject to preliminary descriptive analysis using SPSS statistical software (version 14; SPSS, Chicago, IL, USA) to examine the frequency of independent and dependent variables. The data was then subject to multi-level logistic regression analysis to examine patient and provider factors associated with assessment and intervention for lifestyle risk factors. Multi-level analysis was considered appropriate as patient data was highly clustered by provider for assessment (ICC = 0.386) and intervention (ICC = 0.422). The intra class correlation (ICC) represents the degree to which audit responses for patients seen by the same provider are similar to one another compared with those of patients from different providers. The high ICC values indicate that the analysis must account for the variance between providers, supporting the choice of multi-level analysis [35].

Multilevel logistic regression models were used with two dichotomous dependent variables adjusted for clustering of patients (level 1) within providers (level 2) [36]. This method also has the advantage of allowing the proportion of variability attributable to each level of analysis to be determined [31]. Initially, we fitted a baseline variance component or empty model (no independent variables) for each of the response variables followed by the model with patient and consultation variables (Model 1). The final model (Model 2) expands Model 1 by including provider variables. The significance of the fixed and random parameter variance estimates (provider variance) was assessed using the Wald joint χ^2 ^test statistic [36]. The proportion of the provider level variance explained for each model was estimated as the difference in provider variance between baseline (empty model) and Model 1 or Model 2 divided by the provider variance for the baseline model [37]. ICC was calculated using the latent variable method. The (standard) logistic distribution has variance π^2^/3 = 3.29 and hence this can be taken as the level 1 variance. As both the level 1 and 2 variances are on the same scale, the following formula was used: ICC = (level 2 variance)/(level 2 variance + 3.29) [35]. All multi-level models were performed with MLwiN version 2.0. [36].

### Ethics

The study was approved by the UNSW Human Research Ethics Committee (HREC) and the HREC in each AHS. All participants gave their informed consent to participate in the study.

## Results

A total of 57 providers out of 61 (93.4%) participated in the audit as evidenced by the return of one or more patient audit records. Audit records were returned for 732 patients across the three teams for the two week audit period (the majority coming from team one) with a mean of around 13 patient audit records returned per provider (ranging from 1–36) (Table 1). It was only possible to obtain figures on the total number of patients seen during the audit period for team one who used an electronic clinical information system. Unfortunately in teams two and three consultation data was not being consistently recorded using a single clinical information system (as teams were in the process of changing systems). For team one audit records were provided for 506 of the 743 (68.1%) patients seen during the audit period.

**Table 1:** Number of providers participating in the audit and number of patient audit records returned.

Team	No. providers participating in audit	Total Number of providers in each team	Total number of patients audited	Mean No. patients audited per provider	Range of patients per provider
Team 1	33	35	506	15.3	2–28
Team 2	16	16	147	9.2	1–36
Team 3	8	10	79	9.8	7–14
**Total**	**57**	**61**	**732**	**12.8**	**1–36**

### Patient, Consultation and Provider Characteristics

The majority of patients audited (62%) were over 65 years of age with approximately 20% aged 45–64 years and 18% aged 18–44. There were more females (59%) than males, with a relatively even distribution of patients from areas of low and high deprivation, although fewer patients lived in areas of intermediate deprivation. More than three quarters of patients were being seen in follow up consultations with over half (57%) being seen for wound management. Risk factors were considered appropriate to address with the majority of patients (82%). However risk factors were deemed inappropriate to address in just under one in five patients mainly due to extrinsic patient factors (such as physically unable to respond, frail age, palliative care) or service factors (such as lack of time or discrete/casual service) (Table 2).

**Table 2:** Patient, consultation and provider characteristics

**Variables**	**Categories**	**No (%)**
**Patient Characteristics **(n = 732 patients)		
Age (n = 727)	18–44 years	131 (18.0)
	45–64 years	142 (19.5)
	65+	454 (62.4)
		
Gender (n = 728)	Male	302 (41.5)
	Female	426 (58.5)
		
SEIFA index^1 ^(n = 712)	Most disadvantaged (SEIFA quintile 1–2)	314 (44.1)
	Intermediate Deprivation (SEIFA quintile 3)	120 (16.9)
	Least disadvantaged (SEIFA quintile 4–5)	278 (39.0)
		
**Consultation Characteristics **(n = 732 patients)	First consultation	150 (22.3)
Consultation Type (n = 674)	Follow up consultation	524 (77.7)
		
Reason for Visit (n = 660)	Wound management	377 (57.1)
	Procedures	59 (8.9)
	Primary health care clinic (team 3 only)	56 (8.5)
	Child and Family Health	49 (7.4)
	Palliative care	44 (6.7)
	Chronic disease care	28 (4.2)
	Allied health	25 (3.8)
	Support (unspecified)	22 (3.3)
		
Appropriate to address risk factors (n = 686)	Yes	564 (82.2)
	No – extrinsic patient or service factors^2^	86 (12.5)
	No – patient acceptability low^3^	36 (5.3)
		
**Provider Characteristics **(n = 57 providers)		
		
Team (n = 57)	Team 1 (Urban)	33 (57.9)
	Team 2 (Rural)	16 (28.1)
	Team 3 (Rural)	8 (14.0)
		
Provider Type (n = 57)	Registered Nurse	37 (64.9)
	Enrolled Nurse	11 (19.3)
	Allied Health/other^4^	9 (15.8)
**Provider Attitudes **(n = 57 providers)		
Plan to discuss risk factors prior to consultation (n = 680)	No	395 (58.1)
	Yes	285 (41.9)
		
Accessibility of Support Services Rating (n = 56)	Low	10 (17.9)
	Moderate	33 (58.9)
	High	13 (23.2)
		
Work Priority Rating (n = 56)	Low	5 (8.9)
	Moderate	17 (30.4)
	High	34 (60.7)
		
Patient Acceptability Rating (n = 55)	Low	3 (5.5)
	Moderate	30 (54.5)
	High	22 (40)
		
Effectiveness Rating (n = 55)	Low	11 (19.6)
	Moderate	40 (71.4)
	High	4 (7.1)

^1 ^2006 index of relative socio-economic advantage/disadvantage

^2 ^Extrinsic patient or service factors: Palliative care, physically unable to respond, frail aged, incapacitated, mental health problems, intellectual disability, NESB, lack of time, discrete/casual service, ongoing care

^3 ^Provider perception of low patient acceptance (inappropriate time, patient not interested, religious beliefs)

^4 ^Other: Aboriginal Health Worker (AHW), as only one AHW participated in the audit they have been grouped with allied health providers for the purposes of examining impact of provider type in multi-level analysis.

The majority of providers participating in the audit were registered nurses (n = 37) working in an urban area (team 1, n = 33) with 24 providers working in rural areas (teams 2 and 3). There were also 11 enrolled nurses and eight allied health practitioners and one Aboriginal Health Worker. The majority of providers (61%) rated addressing risk factors to be a high work priority, 40% considering patient acceptability as high. However only one in four rated accessibility of support services as high and very few (7%) rated their effectiveness in helping patients change as good or excellent.

### Assessment and Intervention for Lifestyle Risk Factors

According to the audit records, assessment of lifestyle risk factors occurred in the majority of patients ranging from 61.5% of patients having alcohol intake assessed up to 72% for nutrition assessment (Table 3). A higher proportion of patients were identified to be physically inactive (25.5%) and with poor nutrition (22.4%) compared to those currently smoking (11.1%) and those with 'at-risk' alcohol consumption (4.6%). Most patients with a risk factor recorded where also noted as having received an intervention, ranging from 85.2% of smokers to 91.5% of those with poor nutrition (Table 3).

**Table 3:** Rates of assessment and management for behavioural risk factors

	**No (%) patients with risk factor assessed**	**No (%) patients with risk factor recorded**	**No (%) of patients with risk factor recorded receiving intervention^1^**
**Smoking**	492 (67.2)	81 (11.1)	69 (85.2)
**Nutrition**	527 (72.0)	164 (22.4)	150 (91.5)
**Alcohol**	450 (61.5)	34 (4.6)	29 (85.3)
**Physical Activity**	506 (69.1)	187 (25.5)	164 (87.7)

^1 ^Intervention: verbal advice, written advice, referral in current or previous consultation

The rates of optimal assessment and intervention for lifestyle risk factors are shown in Table 4. Just over half (54.1%) of all patients were assessed for all four lifestyle risk factors (defined as optimal practice) and 45.9% were assessed for only some or none of the risk factors (sub-optimal practice). For patients with at least one lifestyle risk factor recorded, 86.3% received intervention for all identified risk factors (optimal practice) compared to only 13.7% of 'at risk' patients receiving intervention for none or only some of the risk factors identified (sub-optimal practice) (Table 4).

**Table 4:** Rates of optimal assessment and intervention practices for behavioural risk factors

**Variable**	
**Risk Factor Assessment**	**No (%) of all patients (n = 732)**
No assessment for any risk factors	130 (17.8)
Assessment for 1 risk factor	77 (10.5)
Assessment for 2 risk factors	73 (10.0)
Assessment for 3 risk factors	56 (7.7)
**Sub-optimal**: Assessment for 3 or fewer risk factors	336 (45.9)
**Optimal**: Assessment for all four risk factors	396 (54.1)
**Risk Factor Intervention^1^**	**No (%) of patients with at least one risk factor recorded (n = 307)**
No intervention offered for any risk factors identified	29 (9.4)
Intervention offered for some risk factors identified	13 (4.2)
Intervention offered for all risk factors identified	265 (86.3)
**Sub-optimal**: Intervention for none or some of the risk factors identified	42 (13.7)
**Optimal**: Intervention for all of the risk factors identified	265 (86.3)

^1 ^Intervention: verbal advice, written advice, referral in current or previous consultation

### Factors Associated with Optimal Assessment for Lifestyle Risk Factors

Between provider variability for assessment of lifestyle risk factors was high (ICC = 0.386). Table 5 shows two different models for assessment for lifestyle risk factors (optimal practice: assessment of all lifestyle risk factors versus suboptimal practice: assessment of three or less lifestyle risk factors). Variables were included in a stepwise fashion, with Model 1 containing patient and consultation variables and Model 2 including patient, consultation and provider variables. In the final model (Model 2), no patient characteristics were independently associated with assessment practices. However, patients were more likely to be screened for all risk factors in the first consultation compared to a follow up consultation (OR = 3.22, CI = 1.63–6.34, Model 2). Patients being seen for wound management or procedures were also more likely to be assessed for all lifestyle risk factors compared to patients receiving palliative care. No provider variables were significantly associated with assessment practices in the final model after controlling for patient and consultation factors. However two provider attitude variables approached significance. Firstly providers who intended to discuss risk factors prior to the consultation were more likely to assess for all lifestyle risk factors (approaching significance, P = 0.06, OR = 1.70, CI = 0.97–2.97, Model 2). Similarly the perceived accessibility of support services was positively associated with assessment (approaching significance, P = 0.10, OR = 1.45, CI = 0.92–2.28, Model 2).

**Table 5:** Multi-level logistic regression models for assessment for lifestyle risk factors

**Explanatory Variables**		**Empty Model**	**Model 1^1^**	**Model 2^1^**
**Patient Characteristics**		OR (95% CI)	OR (95% CI)	OR (95% CI)
Age	65+ years		1.00 (reference)	1.00 (reference)
	18–44 years		0.86 (0.43–1.73)	1.06 (0.48–2.33)
	45–64 years		1.00 (0.57–1.74)	1.02 (0.56–1.87)
				
Gender	Male		1.00 (reference)	1.00 (reference)
	Female		1.01 (0.66–1.55)	0.99 (0.62–1.60)
				
SEIFA index^2^	Most disadvantaged		1.00 (reference)	1.00 (reference)
	Intermediate disadvantage		0.86 (0.34–2.16)	0.61 (0.22–1.72)
	Least disadvantaged		1.59 (0.65–3.87)	1.28 (0.46–3.53)
				
**Consultation Characteristics**				
Consultation Type	Follow up consultation		1.00 (reference)	1.00 (reference)
	First consultation		**3.19 (1.83–5.58)**	**3.22 (1.63–6.34)**
				
Reason for Visit	Palliative care		1.00 (reference)	1.00 (reference)
	Primary health care clinic		**6.57 (1.56–27.71)**	5.37 (0.85–33.87)
	Wound management		**4.09 (1.72–9.71)**	**4.25 (1.74–10.34)**
	Procedures		**3.03 (1.01–9.04)**	**3.69 (1.16–11.77)**
	Chronic disease care		4.00 (0.84–19.00)	3.10 (0.5–19.33)
	Allied health		0.79 (0.08–8.32)	0.28 (0.01–8.30)
	Child and Family Health		**10.18 (1.51–68.52)**	4.17 (0.38–45.43)
	Support (unspecified)		1.75 (0.36–8.46)	2.00 (0.34–11.83)
				
**Provider Characteristics**				
Team	Team 1		1.00 (reference)	1.00 (reference)
	Team 2			1.06 (0.17–6.43)
	Team 3			1.01 (0.13–7.88)
				
Provider Type	Registered Nurse			1.00 (reference)
	Enrolled Nurse			1.43 (0.37–5.57)
	Allied Health or other			1.35 (0.18–10.03)
				
**Provider Attitudes**				
Plan to discuss risk factors prior to consultation	No			1.00 (reference)
	Yes			1.70 (0.97–2.97)
				
Perceived Accessibility of Support Services				1.45 (0.92–2.28)
Work Priority Rating				0.64 (036–1.15)
Perceived Patient Acceptability				1.18 (0.64–2.17)
Perceived Effectiveness				0.94 (0.51–1.75)
Between provider variance (SE^5^)		2.070 (0.593)	1.896 (0.499)	1.701 (0.488)
Intra class correlation		0.386	0.366	0.341
Explained variance^6 ^(%)		-	8.41%	17.83%

Multilevel logistic regression. Patients, n = 738. ^1 ^Model 1: Patient and consultation variables, Model 2: Patient, consultation and provider variables., ^2 ^2006 index of relative socio-economic advantage/disadvantage, ^3 ^Extrinsic patient or service factors: Palliative care, physically unable to respond, frail aged, incapacitated, mental health problems, intellectual disability, NESB, lack of time, discrete/casual service, ongoing care, ^4^Provider perception of low patient acceptance (inappropriate time, patient not interested, religious beliefs), ^5 ^Standard error, ^6 ^Explained 'between provider' variance using the variance in the empty model as reference.

The between-provider variance (random intercept) was statistically significant in both assessment models, indicating that there were significant differences in assessment for all lifestyle risk factors between providers after adjusting for patient, consultation and provider variables. Patient and consultation variables (Model 1) explained 8% of the between provider variance in assessment, while patient, consultation and provider variables (Model 2) explained approximately 18% of the between provider variance in assessment. This suggests that factors other than those measured here are important in explaining the variation in assessment practices.

### Factors Associated with Providing Optimal Intervention for Lifestyle Risk Factors

A high between provider variability (ICC = 0.422) was also observed for providing intervention for lifestyle risk factors. Table 6 shows two different models for intervention (optimal practice: providing intervention for all lifestyle risk factors identified versus sub-optimal practice: providing intervention for none or only some risk factors identified). As with assessment, variables were included in a stepwise fashion. Model 1 contained patient and consultation variables and Model 2 included patient, consultation and provider variables. In the final model after controlling for the team in which providers worked and other provider variables, the index of relative socio-economic advantage/disadvantage for the area in which the patient lived was significantly associated with intervention practices. The least disadvantaged patients were more likely to receive optimal intervention compared to the most disadvantaged patients (OR = 11.29, CI = 1.23–103.83, Model 2).

**Table 6:** Multi-level logistic regression models for intervening for lifestyle risk factors

**Explanatory Variables**		**Empty Model**	**Model 1^1^**	**Model 2^1^**
**Patient Characteristics**		OR (95% CI)	OR (95% CI)	OR (95% CI)
Age	65+ years		1.00 (reference)	1.00 (reference)
	18–44 years		1.93 (0.43–8.69)	0.98 (0.16–6.06)
	45–64 years		1.55 (0.45–5.37)	2.66 (0.48–14.72)
				
Gender	Male		1.00 (reference)	1.00 (reference)
	Female		0.74 (0.28–1.97)	0.60 (0.17–2.12)
SEIFA index^2^	Most disadvantaged		1.00 (reference)	1.00 (reference)
	Intermediate disadvantage		2.37 (0.43–13.18)	1.53 (0.17–14.00)
	Least disadvantaged		4.11 (0.79–21.46)	**11.29 (1.23–103.83)**
				
**Consultation Characteristics**				
Consultation Type	Follow up consultation		1.00 (reference)	1.00 (reference)
	First consultation		0.79 (0.27–2.27)	0.52 (0.11–2.59)
Reason for Visit	Palliative care		1.00 (reference)	1.00 (reference)
	Wound management, Primary health care clinic & procedures		3.72 (0.71–19.51)	**9.84 (1.21–80.25)**
	Other		2.91 (0.39–21.76)	3.11 (0.19–49.87)
Appropriate to address risk factors	No – extrinsic patient or service factors or patient acceptability low^3^		1.00 (reference)	1.00 (reference)
	Yes		**9.70 (3.23–29.12)**	**25.89 (4.78–140.26)**
**Provider Characteristics**				
	Team 1 (Urban)			1.00 (reference)
	Team 2 & 3 (Rural)			**14.24 (1.55–130.94)**
Provider Type	Nurse			1.00 (reference)
	Allied Health			0.39 (0.05–2.91)
				
**Provider Attitudes**				
Plan to discuss risk factors prior to consultation	No			1.00 (reference)
	Yes			0.66 (0.15–2.96)
Perceived Accessibility of Support Services				**0.25 (0.11–0.59)**
Work Priority Rating				1.77 (0.89–3.53)
Perceived Patient Acceptability				2.32 (0.81–6.62)
Perceived Effectiveness				**0.23 (0.06–0.80)**
Between provider variance (SE^5^)		2.403 (1.079)	1.880 (0.902)	0.459 (0.644)
Intra class correlation		0.422	0.364	0.122
Explained variance^6 ^(%)		-	21.76	80.90

Multilevel logistic regression. Patients, n = 307. ^1 ^Model 1: Patient and consultation variables, Model 2: Patient, consultation and provider variables., ^2 ^2006 index of relative socio-economic advantage/disadvantage, ^3 ^Extrinsic patient or service factors: Palliative care, physically unable to respond, frail aged, incapacitated, mental health problems, intellectual disability, NESB, lack of time, discrete/casual service, ongoing care, ^4^Provider perception of low patient acceptance (inappropriate time, patient not interested, religious beliefs), ^5 ^Standard error, ^6 ^Explained 'between provider' variance using the variance in the empty model as reference.

Similarly, after controlling for providers variables, patients seen for wound management, procedures or in PHC clinics were more likely to have received intervention for all lifestyle risk factors compared with patients receiving palliative care (OR = 9.84, CI = 1.21–80.25, Model 2).

Patients were also significantly more likely to have received lifestyle interventions when providers considered it to be 'appropriate' compared to patients where it was considered inappropriate because of extrinsic patient or service factors such as the patient being of frail age or due to a lack of time (OR = 25.89, CI = 4.78–140.26, Model 2). After controlling for patient and consultation factors, a number of provider attitude variables were also significantly associated with providing optimal intervention. The perceived accessibility of support services was negatively associated with the likelihood of providing risk factor interventions to patients (OR = 0.25, CI = 0.11–0.59, Model 2), as was the perceived effectiveness of intervention (OR = 0.23, CI = 0.06–0.80, Model 2).

The between-provider variance (i.e. the random intercept) was statistically significant in Model 1 but not in Model 2. This indicates that there were significant differences in intervening for lifestyle risk factors between providers after adjusting for patient and consultation variables, however the addition of provider variables explained most (over 80%) of the variation between providers. Overall patient and consultation factors explained 22% of the between provider variance in intervention (Model 1), while patient, consultation and provider variables together explained over 80% of the between provider variance in intervention (Model 2). This suggests that the provider variables included in Model 2 were important in explaining the variation in intervention practices.

## Discussion

This is one of the first studies of its kind examining the relative importance of patient, consultation and providers factors in influencing the assessment and intervention for lifestyle risk factors in PHC outside of general practice. Overall, our results show high variation between providers in assessment and intervention for lifestyle risk factors. Consultation characteristics such as the consultation type and reason for the visit were the most important in explaining the variation in assessment practices, however these factors along with patient and provider variables accounted for less than 20% of the variance. In contrast provider characteristics and attitudes, in particular the team in which providers worked, perceived accessibility of support services and perceptions of effectiveness were the most important in explaining the variation in intervention practices. After controlling for provider variables, patients' socio-economic status, the reason for the visit and providers' perceptions of the 'appropriateness' of addressing risk factors in the consultation were all significantly associated with providing optimal intervention. Together patient, consultation and provider variables accounted for most (80%) of the variation in intervention practices.

Our results suggest that the assessment of lifestyle risk factors is influenced by different factors than those determining intervention practices. Understanding the determinants of assessment is important as our results show that 80–90% of patients with a risk factor identified were recorded to have received an intervention, although this may reflect over-reporting of desirable practices. Screening for all lifestyle risk factors was more likely to occur in first consultations, when the patient presented for wound management and procedures and when providers planned to discuss risk factors prior to the consultation (approaching significance). This is likely to reflect service protocols used by the teams participating in the study, whereby screening for behavioural risk factors is part of the standard assessment process usually undertaken at the first visit.

The final assessment model including all variables was however, only able to explain 18% of the between-provider variance in assessing for lifestyle issues. This may be a result of methodological limitations. The combining of assessment scores across each type of risk factor (smoking, nutrition, alcohol and physical activity) may have prevented the detection of associations. Furthermore over 80% of patients had at least one risk factor assessed, hence low assessment practices were defined for the purposes of multi-level analysis as asking about three or less risk factors while asking about all four risk factors was considered high assessment practices. Another possibility is that assessment practices are influenced by variables other than those measured in this study such as provider workload. Other studies have reported that patient factors such as the number of health problems and the frequency of patient visits is associated with screening for behavioural risk factors [30,38,39]. We were unable to locate any studies that have attempted to explain the variation in assessment practices using patient, provider and consultation factors. Further research is required to understand the determinants of assessment practices.

Unlike the assessment model, the final intervention model was able to explain most (over 80%) of the between provider variance in intervening for lifestyle risk factors. Provider characteristics and attitudes, in particular the team in which the providers worked and beliefs about perceived effectiveness and accessibility of support services, were most important in determining intervention practices (explaining over 60% of the between provider variance), compared to patient or consultations factors (explaining 20% of the between provider variance). Interestingly patients socio-economic status and reason for the visit were only significantly associated with intervention practices after controlling for provider factors. These results suggest the importance of provider factors such as the team location (urban versus rural) in moderating the association between patient and consultation characteristics and intervention practices.

Providers working in rural teams (teams two and three) were more likely to provide optimal intervention than those working in the urban area (team one) after controlling for patient and consultation characteristics and other provider attitudes. This is likely to reflect differences in work practices and priorities of the teams, with team one predominantly focusing on provision of post acute nursing care and the rural teams providing a broader range of services with team three in particular focusing on provision of PHC services. It may also reflect differences in the workload and opportunities available for lifestyle screening and intervention in urban and rural teams.

The relationship between providing optimal intervention and provider attitudes, in particular perceptions about effectiveness of the interventions and provider beliefs about the accessibility of support services warrants further discussion. In line with previous studies, providers in this study were generally pessimistic about the effectiveness of intervening for lifestyle risk factors, with only four providers (7.1%) rating their effectiveness as high across all risk factors [6,40]. Surprisingly the level of perceived effectiveness was negatively associated with the likelihood of providing optimal intervention for all lifestyle risk factors. This finding is not easily explained and is in conflict with previous studies that have reported low levels of perceived effectiveness to be a barrier to the provision of lifestyle intervention [16,41,42]. One explanation may be that providers with higher levels of perceived effectiveness may provide more intensive intervention to fewer more selective patients or focusing on particular lifestyle risk factors. The audit data only provided information about the frequency of intervention rather than intervention intensity. This requires further exploration in future research.

Another provider attitude of interest was the perceived accessibility of support services which was positively associated with screening (approaching significance) but negatively associated with intervention practices. This finding suggests that providers' who perceive that there are few places to refer patients to for help are less likely to screen for lifestyle issues. However once risk factors have been identified providers are more likely to provide optimal intervention themselves if they have limited options for referral. Role expectations in addressing risk factors may be shaped to some extent by the accessibility of other providers or services who can take on this role. This hypothesis was also put forward by Pelletier-Fluery et al, [32] who found that the density of GPs in the community was negatively associated with GP delivery of CVD preventative services. While studies have reported that access to support services for lifestyle modification is often limited and that rates of referral for lifestyle issues is generally low [10,43], we are unaware of other studies that have examined the association between perceived access to support services for lifestyle risk factors and rates of screening and intervention for lifestyle risk factors in PHC. These findings warrant further investigation in future studies.

We also found the consultation context important in determining intervention practices. Patients were more likely to receive optimal intervention when attending for wound management, procedures and PHC clinics and when providers considered it appropriate to address lifestyle issues. This is line with a number of other studies that have found that PHC providers are more likely to address lifestyle issues during 'wellness' or chronic care consultations [18,20,44]. This may reflect greater priority placed on addressing lifestyle issues in these consultations, more opportunity and/or higher perceived patient acceptance. This finding highlights the importance of the service delivery context in particular opportunities for continuity of care, consultations dedicated to health assessment and monitoring that facilitate the provision of lifestyle interventions.

The only patient characteristic significantly associated with intervention practices was patients' socioeconomic status. After controlling for provider factors the least disadvantaged patients were more likely to receive optimal intervention compared to the most disadvantaged patients. This is an important finding given the higher prevalence of chronic diseases and lifestyle risk factors amongst lower socio-economic groups [45]. Qualitative data collected in the larger feasibility study suggest that providers may be reluctant to offer intervention to disadvantaged patients with more pressing problems, as lifestyle habits such as smoking are considered to be the patients 'crutch' and these patients are perceived to have less capacity and motivation to change. [46]. The association between indicators of socioeconomic status and receipt of lifestyle interventions in previous studies is mixed. Studies reporting on recall of lifestyle advice by patients have found that receiving advice is significantly higher for patients with lower levels of education [20,22]. In contrast studies examining intervention practices measured or observed in individual encounters have found unskilled and lower educated patients to be less likely to receive lifestyle intervention [24,27]. Others have found no association with socioeconomic status [28,29,44]. To our knowledge no previous studies have taken into account provider characteristics such as location of team or practice which may confound the association between socioeconomic status and intervention practices. This is clearly an area requiring further research.

The study has a number of limitations. Firstly our findings are based on a relatively small sample of PHC providers working in a small number of community health teams. It is not certain how these findings would apply to PHC workers in other settings. Teams were selected based on an expression of interest, hence may have been more interested and motivated to address lifestyle risk factors compared to other teams. Data was however collected from 57 of 61 (93%) of providers in the nominated teams, avoiding self selection of interested practitioners. The audit was undertaken prospectively and based on self-report, which may not reflect actual practice and could lead to over-reporting of activities perceived as socially desirable. The high rates of optimal assessment and intervention reported in this study suggest that over-reporting is probable. The findings related to lifestyle intervention should also be interpreted with caution due to the small number of cases with sub-optimal practice and the relatively large number of variables included in the analysis. It was also not possible to determine the response rate for the proportion of patients audited over the 2 week period in teams two and three and providers may have audited selective patients where risk factor management practices were favourable. As the audit was undertaken prospectively and not recorded during the consultations and this may have influenced the recall and accuracy of the recording of risk factor management practices. Finally it is uncertain if there are differences in the determinants of practice for each type of risk factor for example smoking cessation counselling versus nutrition.

## Conclusion

In conclusion, our results show considerable variation between PHC providers in the rate at which they assess and offer intervention for lifestyle risk factors. The findings highlight the importance of provider factors including beliefs and attitudes, team location (urban versus rural) and consultation context in determining rates of intervention for lifestyle risk factors, along with patients' socio-economic status. The variation between providers in undertaking assessment for lifestyle issues was not well explained by the variables measured in this study. Further research is required to improve our understanding of the key determinants of assessment and intervention practices for lifestyle risk factors by PHC providers. The inclusion of organisational factors such as service protocols and workload may improve the variance explained in future models. Multi-level models provide a useful means for determining the relative importance of patient versus provider/organisational factors in influencing the management of behavioural risk factors.

## Competing interests

The authors declare that they have no competing interests.

## Authors' contributions

RAL contributed to study design, data collection, descriptive quantitative analysis and wrote the first draft of the manuscript. UWJ undertook the multi-level logistic regression analysis contributed to data interpretation and writing of the manuscript. MFH contributed to study design, data analysis and interpretation. AMW and GPD contributed to study design and data interpretation. LAK contributed to data analysis and interpretation. All authors read and approved the final manuscript.

## Appendix 1

Below is a description of the role of various community health providers involved in the project.

### Registered and Enrolled Generalist Community Nurses

In Australia registered nurses undertake a three year tertiary education program. Enrolled nurses undertake training from 12 months to two years at a technical college receiving a certificate or diploma depending on the state. Enrolled nurses work with registered nurses to provide patients with basic nursing care. Within the project registered nurses undertook the initial patient assessment and care planning and enrolled nurses assisted with the implementation of the care plan. In team one, generalist community nursing predominately conducted home visits and saw patients for wound management, medication administration, chronic disease management and palliative care. The majority of patients were referred following discharge from hospital and were over 65 years of age. In team two, each of the 'generalist' community nurses had a specific role such as diabetes education, cardiac rehabilitation, women's health, palliative care, disabilities and aged care. Most patients were seen on an individual basis, either through home visits or at clinics held at the community health centre.

### Primary Health Care (PHC) Nurses

PHC nurses in team three were all registered nurses. Each PHC nurse worked with a number of rural or remote communities providing regular 'drop in' clinics focusing on health screening and monitoring which were held at community venues such as the local community hall or church. PHC nurses in this team also reported spending at least half their time conducting community education and development activities such as undertaking needs assessment of local communities, providing group education programs for adults, health education programs in schools and health screening at community venues or events.

### Child and Family Nurses

Child and family health nurses are registered nurses or midwives (often with graduate certificate or graduate diploma level qualifications in maternal, child and family health) who provide a range of services to families with infants and young children. This includes health education and promotion in the form of baby clinics, parenting groups, individual patient care and primary school involvement. Services may vary from state to state and territory but the goals are similar: to provide infants, children and adolescents and their families with a range of professional services that have a strong emphasis on prevention, early intervention, support and referral to other services.

### Aboriginal Health Worker (AHW)

AHWs provide clinical and primary health care for individuals, families and community groups. This includes health screening, monitoring and community development work as well as liaising between the Aboriginal community and other health professionals. Most AHWs undertake a certificate III or IV in Aboriginal Primary Health Care Work at a technical college, however educational requirements vary nationally. In this project, AHWs in team three worked along side PHC nurses in a number of rural and remote communities providing a combination of health screening and monitoring and undertaking community development activities.

### Allied Health Practitioners

Allied health practitioners working in team one included a social worker, psychologist, speech pathologist, occupational therapist and dietitian. Team three had two allied health practitioners: a social worker and counsellor.

## Pre-publication history

The pre-publication history for this paper can be accessed here:



## Supplementary Material

Additional file 1 **Findings of preliminary analysis to create aggregate assessment variable for multi-level analysis**. The data provided represent the preliminary statistical analysis undertaken to create aggregate assessment variable for multi-level analysis. Click here for file
