# Supplementary material for: Explaining the variation in the management of lifestyle risk factors in primary health care: A multilevel cross sectional study
Source: BMC Public Health. 2009 May 29;9:165. doi: 10.1186/1471-2458-9-165 (PMC2698853; doi:10.1186/1471-2458-9-165)
Supplement: Additional file 1 — Findings of preliminary analysis to create aggregate assessment variable for multi-level analysis. The data provided represent the preliminary statistical analysis undertaken to create aggregate assessment variable for multi-level analysis. [file 1471-2458-9-165-S1.doc]

# Additional File 1: Findings of preliminary analysis to create aggregate assessment variable for multi-level analysis

Preliminary analysis was undertaken to determine whether it would be possible to aggregate assessment variables across risk factors. The findings of this preliminary analysis are provided below:

***Assessment Variables:***

Assessment for smoking was found to be significantly associated with alcohol assessment (Table 1), similarly nutrition assessment was significantly related physical activity assessment (Table 2). As a result two new variables were created 1) ‘assessment for addictions’ which was produced by summing the variables ‘smoking assessment’ and ‘alcohol assessment’ and 2) ‘nutrition and physical activity assessment’ which was produced by summing variables ‘nutrition assessment’ and ‘physical activity assessment’. Finally cross tabulation that these two variables were significantly associated (Table 3). As a result a single assessment variable was created by summing all assessment variables together. The resulting variable had scores ranging from 0= no assessment for any risk factors, 1 = assessment for one risk factor, 2 = assessment for two risk factors, 3= assessment for three risk factors and 4= assessment for all four risk factors. As this assessment variable was not normally distributed (Figure 1) it was recoded into a dichotomous variable with 0= assessment for three or less risk factors and 1= assessment for all four risk factors for use in multi-level analysis.

**Table 1. Cross tabulation of assessment for smoking and alcohol**

|  | |  | **Alcohol Assessment** | | **Total** |
| --- | --- | --- | --- | --- | --- |
|  | |  | **no** | **yes** |  |
| **Smoking Assessment** | **no** | **Count** | **226** | **14** | **240** |
|  |  | % within smoking assessment | 94.2% | 5.8% | 100.0% |
|  |  | % within alcohol assessment | 80.1% | 3.1% | 32.8% |
|  | **yes** | **Count** | **56** | **436** | **492** |
|  |  | % within smoking assessment | 11.4% | 88.6% | 100.0% |
|  |  | % within alcohol assessment | 19.9% | 96.9% | 67.2% |
| **Total** | | **Count** | **282** | **450** | **732** |
|  | | % within smoking assessment | 38.5% | 61.5% | 100.0% |
|  | | % within alcohol assessment | 100.0% | 100.0% | 100.0% |

Continuity Correction (2 x 2 table): X^2^ = 463.30 (df=1), P=0.000

**Table 2. Cross tabulation of screening for nutrition and physical activity**

|  | |  | **Physical activity** assessment | | **Total** |
| --- | --- | --- | --- | --- | --- |
|  | |  | **no** | **yes** |  |
| **Screening for nutrition** | **no** | **Count** | **157** | **48** | **205** |
|  |  | % within nutrition assessment | 76.6% | 23.4% | 100.0% |
|  |  | % within Physical activity assessment | 69.5% | 9.5% | 28.0% |
|  | **yes** | **Count** | **69** | **458** | **527** |
|  |  | % within nutrition assessment | 13.1% | 86.9% | 100.0% |
|  |  | % within Physical activity assessment | 30.5% | 90.5% | 72.0% |
| **Total** | | **Count** | **226** | **506** | **732** |
|  | | % within nutrition assessment | 30.9% | 69.1% | 100.0% |
|  | | % within Physical activity assessment | 100.0% | 100.0% | 100.0% |

Continuity Correction (2 x 2 table): X^2^ = 275.81 (df=1), P=0.000

**Table 3. Cross tabulation of screening for ‘smoking and alcohol’ with screening for**

**‘nutrition and physical activity’**

|  | |  | **nutrition and PA assessment** | | | **Total** |
| --- | --- | --- | --- | --- | --- | --- |
|  | |  | **no assessment** | **nutrition or PA assessment** | **nutrition and PA assessment** |  |
| **screening for smoking & alcohol** | **no screening** | **Count** | **130** | **59** | **37** | **226** |
|  |  | % within smoking & alcohol assessment | 57.5% | 26.1% | 16.4% | 100.0% |
|  |  | % within nutrition and PA assessment | 82.8% | 50.4% | 8.1% | 30.9% |
|  | **smoking or alcohol** assessment | **Count** | **18** | **27** | **25** | **70** |
|  |  | % within smoking & alcohol assessment | 25.7% | 38.6% | 35.7% | 100.0% |
|  |  | % within nutrition and PA assessment | 11.5% | 23.1% | 5.5% | 9.6% |
|  | **smoking and alcohol** assessment | **Count** | **9** | **31** | **396** | **436** |
|  |  | % within smoking & alcohol assessment | 2.1% | 7.1% | 90.8% | 100.0% |
|  |  | screening for nutrition and PA assessment | 5.7% | 26.5% | 86.5% | 59.6% |
| **Total** | | **Count** | **157** | **117** | **458** | **732** |
|  | | % within smoking & alcohol assessment | 21.4% | 16.0% | 62.6% | 100.0% |
|  | | % within nutrition and PA assessment | 100.0% | 100.0% | 100.0% | 100.0% |

Pearson Chi Square: X^2^ = 413.20 (df=4), P=0.000

PA: Physical activity

0= no screening for any risk factors, 1 = screening for one risk factor, 2 = screening for two risk factors, 3= screening for three risk factors and 4= screening for all four risk factors.
